# Comparative evaluation of AlphaFold2 and disorder predictors for prediction of intrinsic disorder, disorder content and fully disordered proteins

**DOI:** 10.1016/j.csbj.2023.06.001

**Published:** 2023-06-02

**Authors:** Bi Zhao, Sina Ghadermarzi, Lukasz Kurgan

**Affiliations:** a Genomics program, College of Public Health, University of South Florida, Tampa, FL, United States; b Department of Computer Science, Virginia Commonwealth University, Richmond, VA, United States

**Keywords:** Intrinsic disorder, Intrinsically disordered protein, AlphaFold2, Prediction, Deep learning, Disorder content, Fully disordered proteins

## Abstract

We expand studies of AlphaFold2 (AF2) in the context of intrinsic disorder prediction by comparing it against a broad selection of 20 accurate, popular and recently released disorder predictors. We use 25% larger benchmark dataset with 646 proteins and cover protein-level predictions of disorder content and fully disordered proteins. AF2-based disorder predictions secure a relatively high Area Under receiver operating characteristic Curve (AUC) of 0.77 and are statistically outperformed by several modern disorder predictors that secure AUCs around 0.8 with median runtime of about 20 s compared to 1200 s for AF2. Moreover, AF2 provides modestly accurate predictions of fully disordered proteins (F1 = 0.59 vs. 0.91 for the best disorder predictor) and disorder content (mean absolute error of 0.21 vs. 0.15). AF2 also generates statistically more accurate disorder predictions for about 20% of proteins that have relatively short sequences and a few disordered regions that tend to be located at the sequence termini, and which are absent of disordered protein-binding regions. Interestingly, AF2 and the most accurate disorder predictors rely on deep neural networks, suggesting that these models are useful for protein structure and disorder predictions.

## Graphical Abstract

**Figure:**
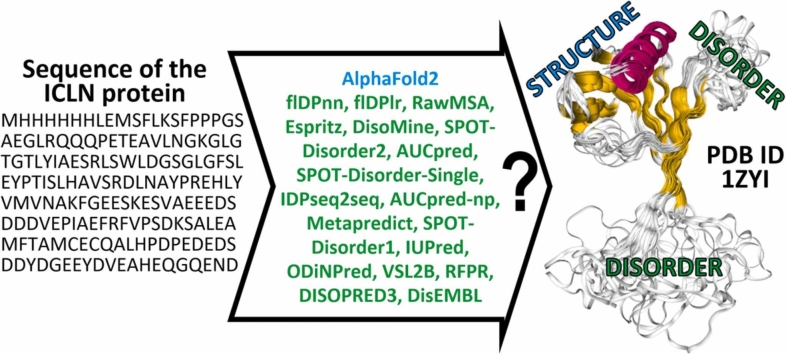

## 1 Introduction

Intrinsically disordered proteins (IDPs) include one or more intrinsically disordered region(s) (IDR) that are absent of a well-defined equilibrium structure under physiological conditions [1], [2], [3]. Bioinformatics studies suggest that IDPs are relatively common in nature, with about a third of eukaryotic proteins that have long IDRs, which are defined as regions with over 30 consecutive amino acids [4], [5], [6], [7]. IDPs contribute to many cellular functions, such as signaling, transcription, translation, molecular assembly, molecular recognition, cell cycle regulation, formation of membraneless organelles, and many others [8], [9], [10], [11], [12], [13], [14], [15]. They are also found across several cellular compartments [16], [17]. The sequences and amino acids that form IDRs have specific/intrinsic biases including depletion in aromatic and bulky hydrophobic amino acids, enrichment in polar and charged residues, and low compositional complexity [18], [19], [20], [21], [22], [23]. These biases make intrinsic disorder predictable from protein sequences. Consequently, many sequence-based computational predictors of intrinsic disorder were developed over the last few decades, with the first method that was published in 1979 [24]. Well over 100 disorder predictors were developed so far [25], [26], [27], [28], [29]. The disorder prediction community recently organized and published a large-scale comparative assessment of predictors, the Critical Assessment of protein Intrinsic Disorder prediction (CAID) experiment [30]. It comparatively evaluated 43 methods concluding that some of the more recently released tools produce relatively accurate results. In particular, CAID and a subsequent empirical analysis found that deep natural network-based methods produce the most accurate results and outperform other types of predictive models [31].

Parallel to these efforts, significant work has been done to develop and advance methods that predict protein structure from sequences. Arguably the key event that measures progress in the structure prediction field is the biennial Critical Assessment of techniques for protein Structure Prediction (CASP) experiment. CASP14, which is the most current published edition, showed that AlphaFold2 (AF2) provides a breakthrough by generating high quality structure predictions [32], [33]. This tool relies on a sophisticated deep network architecture that takes advantage of multiple sequence alignments [34], [35]. The impact of AF2 was further amplified by the release of the database of AlphaFold2-predicted structures, AlphaFoldDB [36], [37]. The most recent version of this resource provides access to the structure predictions for over 214 million proteins, covering nearly the entire UniProt repository [38]. Interestingly, there are several databases of the intrinsic disorder predictions that also include millions of proteins [39], such as Database of Disorder Protein Predictions (D2P2) [40], MobiDB [41], and DescribePROT [42].

A recent commentary uses a popular movie analogy to characterize the AF2 predictions as “*the good, the bad and the ugly*”, which correspond to the majority of accurate predictions, some poor quality predictions, and the ugly predictions for the sequences of IDRs, respectively [43]. While AF2 cannot reliably predict “structures” of IDRs since they are devoid of well-defined structures, an interesting question is whether it can accurately identify where the disordered regions are in an input protein sequence, which is the objective of the disorder predictors. A few studies looked into this question and two alternative approaches were devised to produce scores that can be used to predict IDRs using outputs generated by AF2. The first approach was proposed in the AF2 article and it relies on predicted local distance difference test (pLDDT) values, the per-amino acids confidence scores output directly by AF2 [35]. The second way takes advantage of a previously made observation that the disordered regions have substantially larger surface area compared to the structured regions [44]. Consequently, a few subsequent works use relative solvent accessibility (RSA) generated from the AF2-predicted structure to identify IDRs [45], [46], [47].

We summarize three studies that quantify predictive quality of the AF2-derived scores for the disorder prediction in Table 1. The first study applied AF2 to predict disorder and for several other tasks including prediction of ligand binding sites and structures of protein complexes [45]. The authors showed that the AF2-based disorder predictions are better than the results from a popular disorder predictor, IUPred2, but they did not include other more accurate disorder predictors [30]. Two subsequent studies performed broader analyses [46], [47]. They used the main test dataset from the recent CAID experiment [46], which was collected from the DisProt database [48], and compared AF2 to 8 and 10 disorder predictors that secured accurate results in CAID. Both studies concluded that AF2-based disorder predictions are relatively accurate, however, some disorder predictors outperform the AF2-generated results.

**Table 1:** Comparison of studies that investigate use of AF2 for the intrinsic disorder prediction.

**Reference**	**Considered types of AF2-based disorder predictions**	**Number of considered disorder predictors**	**Include predictors published after CAID was completed (number of these methods)**	**Scope of evaluation**
This study	pLDDT-based and RSA-based	20	Yes (4)	Residue-level disorder, protein-level disorder content, protein level fully disordered proteins, runtime
[45]	pLDDT-based and RSA-based	1	No (0)	Residue level disorder
[46]	pLDDT-based	10	No (0)	Residue level disorder
[47]	pLDDT-based and RSA-based	8	No (0)	Residue level disorder

While the three studies listed in Table 1 provide useful observations, they also share a number of drawbacks that we address. We compare AF2 against a much larger collection of 20 disorder predictors that include the best tools based on the CAID experiment [30], which were also included in the past studies [45], [46], [47], several popular/highly-cited approaches, and a selection of recent methods that were published after CAID ended. We consider both the RSA-based and the pLDDT-based disorder predictions for AF2 while one of the past studies did not utilize the RSA-based approach [46] that was shown to outperform the pLDDT-based disorder predictions [47]. Moreover, we extend scope of the three published articles that focused on the residue-level disorder predictions by additionally covering protein-level predictions of disorder content (i.e., the overall fraction of disordered residues in the protein sequence) and fully disordered proteins. The latter two aspects are commonly evaluated in the disorder prediction field [30], [49], [50], [51]. Furthermore, we investigate several other practical aspects, such as runtime and predictive performance for specific types of IDPs including those with short IDRs, long IDRs, and binding IDRs. We also formulate criteria that identify proteins for which AF2 predicts disorder more accurately vs. proteins where current disorder predictors excel.

## 2 Materials and methods

### 2.1 Datasets

We benchmark AF2 and disorder predictors on the complete main test dataset from the CAID experiment with 646 proteins that relies on the disorder annotations from DisProt [30]. We downloaded the disorder annotations from https://idpcentral.org/caid/data/1/reference/disprot-disorder.txt and the disordered binding annotations from https://idpcentral.org/caid/data/1/reference/disprot-binding.txt; we use the latter to identify binding IDRs. Two most recent evaluations of AF2-based disorder predictions also sourced their test data from the CAID assessment [46], [47]. However, one of them utilized 475 of the 646 test proteins (74%) while the second is limited to 489 of 646 test proteins (76%). We use 646 protein sequences with 336,595 residues, among which there are 831 IDRs that are composed of 54,604 disordered residues, and 255 binding IDRs containing 21,294 disordered binding residues.

**Fig. 1:**
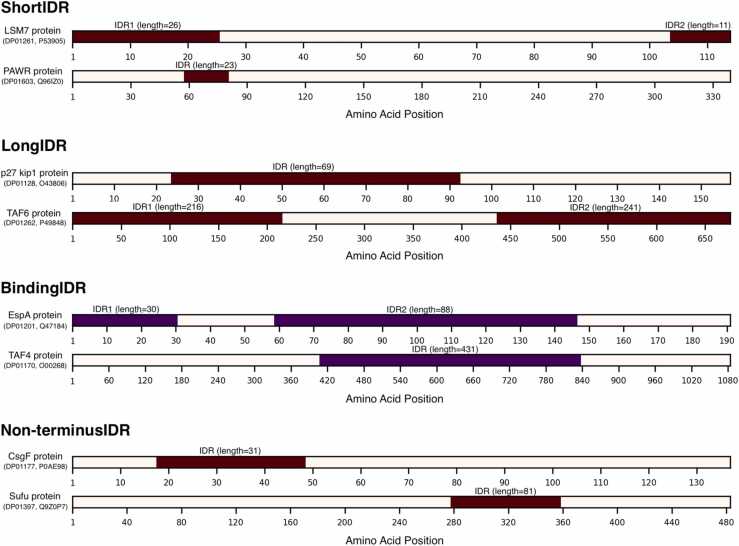
Illustrative examples of proteins that represent the four types of intrinsically disordered proteins: shortIDR (i.e., have only short IDRs), longIDR (have at least one long IDR), bindingIDR (have at least one binding IDR); and non-terminusIDR (do not have IDRs at the sequence termini). We identify proteins by their DisProt and UniProt identifiers. We draw IDRs as brown (for non-binding) and purple (for binding) segments.

We apply these data to create a few additional datasets that include IDPs with specific types of IDRs selected based on their size, location, and function. We separate IDRs by size into long regions (>30 consecutive residues) vs. short regions (≤30). This threshold was used in past works [4], [49], and it roughly divides IDRs into those that are long enough to correspond to protein domains [52] vs. shorter region that may serve as linkers or loops in folded proteins [49]. We also consider location of IDRs in the sequence, in particular separating IDRs that are at the sequence termini vs. those inside the protein chain, given their different functional roles [53]. Moreover, we identify a functional subclass of IDRs that are involved in binding to partner molecules, which is often accompanied by binding induced folding [54], [55], [56]. Correspondingly, we develop four datasets that include IDPs that have: 1) only short IDRs (shortIDR); 2) at least one long IDR (longIDR); 3) at least one binding IDR (bindingIDR); and 4) no IDRs at the sequence termini (non-terminusIDR). The binding IDRs are annotated at the region level, which means that an entire IDR is annotated as binding even if only some of its residues interact with a ligand. This is consistent with the annotations in DisProt and the CAID experiment [30], [48]. We illustrate a few examples that represent these four types of disordered proteins in Fig. 1. We also establish collections of fully disordered proteins (FDPs) following the approach from the CAID experiment [30], i.e., assuming that proteins with a high disorder content set at few different cut-offs (99%, 90%, and 80%) are fully disordered (i.e., FDP99, FDP90, and FDP80 datasets, respectively). We summarize the resulting datasets in Table 2.

**Table 2:** Datasets that we use in the comparative analysis.

**Dataset name**	**Number of proteins**	**Number of IDRs**	**Number of disordered residues**	**Median IDR length**
CAID	646	831	54,604	34
shortIDR	226	281	5062	17
longIDR	420	550	49,542	61
bindingIDR	231	285	23,366	54
non-terminusIDR	318	387	17,971	26
FDP99	45	45	7201	136
FDP90	49	49	8041	138
FDP80	56	57	8986	132

### 2.2 Disorder predictions

We compare predictions generated by AF2 with 20 disorder predictors that we identified in three complementary ways. First, we include the top 10 disorder predictors from the CAID experiment [30] (in alphabetical order): AUCpreD [57], AUCpreD-np[57], DisoMine[58], EspritzD[59], flDPlr[60], flDPnn[60], RawMSA[61], SPOT-Disorder1[62], SPOT-Disorder2[63], and Single-Disorder-Single[64]. Second, we include six most cited predictors based on the citation analysis from [65] (in alphabetical order): DisEMBL-465[66], DisEMBL-HL[66], DISOPRED3[67], IUPred-short[68], IUPred-long[68], and VSL2B[69]. Third, we include four predictors that were released after CAID experiment was completed: ODiNPred [70], IDPseq2seq [71], Metapredict [72], and RFPR[73]. We collected predictions of the top 10 predictors and the six most cited predictors directly from the CAID assessment data at https://idpcentral.org/caid/data/1/predictions/. We used webservers and standalone programs provided by the authors to collect predictions of the four most recent tools.

### 2.3 AlphaFold2 predictions

The two published studies relied on the pre-computed AF2′s predictions from the AlphaFoldDB database, which caused their partial coverage at about 75% of the CAID dataset [46], [47]. We collected prediction directly by using the standalone AF2 software. This allowed us to improve the coverage and measure the AF2 runtime. We were able to make predictions for 632 proteins out of the 646 proteins in the CAID dataset (98% coverage). As we discuss in the introduction, there are two ways to use the AF2′s predicted structure to compute residue-level scores that can be used for the disorder prediction. The original approach introduced by the authors of AF2 is to use the pLDDT values. Correspondingly, we define AF2-pLDDT disorder prediction as 1−pLDDT/100. The other way, which we call AF2-RSA, relies on the RSA values computed from the putative structure that are processed using a sliding window of size 25 [47]. The RSA is calculated by normalizing the DSSP calculated solvent accessibility using the maximum accessibility of a fully extended Gly-X-Gly peptide [74]. We use the implementation from https://github.com/BioComputingUP/AlphaFold-disorder to generate AF2-RSA disorder prediction.

The outputs of AF2 include five ranked structure predictions. We consider two scenarios: the default scenario where we use the top-ranked prediction vs. the optimized-rank scenario where we use one of the five structure models that produces the most accurate disorder prediction. The second scenario simulates a hypothetical prediction where the AF2′s models would be optimally re-ranked to maximize quality of the disorder predictions.

The experimental annotations of disorder from CAID, the AF2-pLDDT and AF2-RSA disorder predictions, and the results produced by the 20 disorder predictors are available in Supplementary Dataset S1.

### 2.4 Metrics for the evaluation of disorder predictions

Disorder predictions include two values that are generated for each amino acid in an input protein sequence: real-valued propensities and binary scores. The latter categorize residues as either disordered or ordered, and they are typically derived from the propensities using a cut-off, i.e., residues with propensities above the cut-off are predicted as disordered and otherwise they are predicted as ordered. We apply metrics that were utilized in recent disorder prediction assessments to evaluate predictions for both output types [27], [30], [31], [47], [75]. We assess the putative propensities with two popular metrics: area under receiver operating characteristic curve (AUC) and area under the precision-recall curve (AUPRC). We generate the binary predictions from the propensities produced by each predictor using a threshold that results in the correct number of disordered residues over the entire CAID dataset. This adequately calibrates the binary predictions between methods and facilitates direct comparisons. These thresholds are listed in Table 3. We evaluate binary predictions using several measures including Matthew correlation coefficient (MCC), F1, and sensitivity:

$$F1=\frac{2*\mathrm{TP}}{2*\mathrm{TP}+\mathrm{FP}+\mathrm{FN}}$$

$$\mathrm{MCC}=\frac{\mathrm{TP}*\mathrm{TN}-\mathrm{FP}8\mathrm{FN}}{\sqrt[{2}]{\left(\mathrm{TP}+\mathrm{FP}\right)*\left(\mathrm{TP}+\mathrm{FN}\right)*\left(\mathrm{TN}+\mathrm{FP}\right)*(\mathrm{TN}+\mathrm{FN})}}$$

$$\mathrm{sensitivity}=\frac{\mathrm{TP}}{\mathrm{TP}+\mathrm{FN}}$$

where TP and TN are the numbers of correctly predicted disordered and structured residues, respectively; FN is the number of disordered residues incorrectly predicted as structured residues; and FP is the number of structured residues incorrectly predicted as disordered residues. We use AUC, AUPRC, MCC and F1 to evaluate the residue-level disorder predictions.

**Table 3:** Comparative assessment for the residue-level disorder predictions on the CAID dataset. We sort predictors by the area under receiver operating characteristic curve (AUC) values. The “Threshold” column provides the cut-off values that we use to convert the real-valued propensities into the binary scores. We assess statistical significance of the differences when compared with the top-ranked flDPnn, AF2-RSA, and AF2-pLDDT, which we highlight using bold font; we show results next to the measured metric using the x|y|z format, where x denotes that flDPnn is significantly better (+), worse (-), and not different (=) than the results from a given predictor at *p*-value = 0.05; y compares against AF2-RSA; and z compares against AF2-pLDDT. Acronyms: area under the precision-recall curve (AUPRC) and Matthew correlation coefficient (MCC).

**Predictor**	**Threshold**	**Coverage (%)**	**AUC**	**AUPRC**	**MCC**	**F1**
**flDPnn**	**0.337**	**100**	**0.814 /-/-**	**0.475 /-/-**	**0.358 /-/-**	**0.462 /-/-**
flDPlr	0.417	100	0.793 + /-/-	0.422 + /-/-	0.323 + /- /-	0.433 + /-/-
AF2-RSAoptimized-rank	0.857	98	0.785 + /-/-	0.357 + /-/-	0.248 + /= /-	0.380 + /= /-
RawMSA	0.683	100	0.780 + /= /-	0.414 + /-/-	0.288 + /= /-	0.404 + /-/-
Espritz-D	0.477	100	0.774 + /= /-	0.410 + /-/-	0.289 + /-/-	0.406 + /-/-
**AF2-RSA**	**0.847**	**98**	**0.768 + / /-**	**0.325 + / /-**	**0.203 + / /-**	**0.343 + / /-**
DisoMine	0.563	100	0.765 + /= /-	0.388 + /- /-	0.244 + /= /-	0.367 + /= /-
SPOT-Disorder2	0.824	94	0.760 + /= /-	0.340 + /= /-	0.200 + /= /-	0.351 + /= /-
AUCpred	1.000	100	0.757 + /= /-	0.479 = /-/-	0.258 + /= /-	0.399 + /-/-
SPOT-Disorder-Single	0.764	100	0.757 + /= /-	0.318 + /= /-	0.221 + /= /-	0.348 + /= /-
IDPseq2seq	0.976	100	0.754 + /= /-	0.322 + /= /-	0.209 + /= /-	0.339 + /= /-
AUCpred-np	1.000	100	0.751 + /+ /-	0.428 + /-/-	0.226 + /= /-	0.349 + /= /-
Metapredict	0.615	100	0.746 + /+ /-	0.340 + /= /-	0.241 + /= /-	0.365 + /= /-
SPOT-Disorder1	0.945	100	0.744 + /+ /-	0.268 + /+ /=	0.143 + /+ /=	0.284 + /= /=
IUPred-short	0.613	100	0.739 + /+ /=	0.311 + /= /-	0.221 + /= /=	0.349 + /= /-
AF2-pLDDToptimized-rank	0.657	99	0.737 + /+ /=	0.289 + /= /=	0.160 + /= /=	0.290 + /= /=
IUPred-long	0.719	100	0.737 + /+ /=	0.298 + /= /=	0.218 + /= /-	0.346 + /= /-
ODiNPred	0.996	100	0.734 + /+ /=	0.314 + /+ /=	0.207 + /= /-	0.330 + /= /-
VSL2B	0.905	100	0.732 + /+ /=	0.301 + /= /-	0.203 + /= /-	0.333 + /= /-
**AF2-pLDDT**	**0.628**	**99**	**0.722 + /+ /**	**0.272 + /+ /**	**0.137 + /+ /**	**0.278 + /+ /**
RFPR	1.000	100	0.721 + /+ /=	0.338 + /= /-	0.109 + /+ /=	0.219 + /+ /+
DISOPRED3	0.965	100	0.701 + /+ /+	0.290 + /= /=	0.122 + /+ /=	0.263 + /+ /=
DisEMBL-465	0.533	100	0.685 + /+ /+	0.283 + /+ /=	0.196 + /+ /-	0.328 + /= /-
DisEMBL-HL	0.131	100	0.654 + /+ /+	0.274 + /+ /=	0.170 + /+ /=	0.302 + /= /=

We follow CAID and use the F1 and sensitivity metrics to evaluate the protein-level predictions of the fully disordered proteins, i.e., application of disorder predictors to identify whether a given sequence is fully disordered or not. The protein-level predictions of disorder content (% of disordered residues in the protein) require separate metrics since both the prediction and the native annotation are real-valued. We compute the native content values as the fraction of the native disordered residues in a given protein chain. We calculate the predicted content values as the fraction of the binary predictions of disorder that are established using the calibration cut-off. We follow past research on the disorder and secondary structure content prediction [51], [76], [77] and use the Mean Squared Error (MAE) and the Spearman Correlation Coefficients (SCC) to quantify the accuracy of the disorder content predictions:

$$\mathrm{MAE}=\frac{1}{n}\sum_{i=1}^{n}|x_{i}-a_{i}|$$

$$\mathrm{SCC}=1-\frac{6\sum_{i}d_{i}^{2}}{n(n^{2}-1)}$$

where *n* is the number of proteins in the dataset, *a*_*i*_ are the native content values, *x*_*i*_ are the predicted content values, and d_i_ is the difference of ranks between the predicted and the native content values. We opt to apply SCC rather than the Person correlation coefficient since the latter is more susceptible to outliers.

### 2.5 Statistical analysis

We assess statistical significance of differences in predictive performance between disorder predictions generated by different methods. In particular, we compare all predictors against the top-ranked method, AF2-pLDDT and AF2-RSA. These tests aim to evaluate robustness of the differences over different datasets, which is why we compare results using several different subsets of the test datasets, which are either disjoint or have a small overlap. For the residue-level tests and the protein-level disorder content tests on the CAID, shortIDR, longIDR, bindingIDR, and non-terminusIDR datasets we perform significance tests using 20 disjoint set of 5% proteins, selected at random. The protein-level assessment for the prediction of fully disordered proteins has smaller number of positive samples (between 45 and 56 fully disordered proteins), which is why we use a larger sampling rate to be able to reliably estimate predictive quality. Thus, we use 20 sets of 20% proteins for the assessments on the FDP99, FDP90, and FDP80 datasets. We perform paired *t*-test (using the same sampled datasets) if the underlying measurements are normal; otherwise, we use the Wilcoxon rank test. We test normality with the Anderson-Darling test at the *p*-value of 0.05.

## 3 Results and discussion

### 3.1 Comparative evaluation of the residue-level disorder predictions

This is a typical assessment scenario that was considered by the past studies that evaluated AF2 [46], [47]. We compare AF2-pLDDT and AF2-RSA against the 20 disorder predictors that cover the best performers in CAID, popular methods and recently published tools. Table 3 summarizes these results.

We observe that AF2-RSA performs significantly better that AF2-pLDDT across the four metrics (*p*-value<0.05). Similar observation was made in ref. [47], although without assessing statistical significance of the differences. The pLDDT scores estimate the degree of agreement between the predictions and the experimental structure and so they could indicate that prediction is poor because the corresponding part of the “structure space” is not accurately covered by the deep network model or because that part of the sequence is disordered. On the other hand, unusually high solvent accessibility implies lack of structure, which seems to be a better proxy for the intrinsic disorder.

The optimized rank version of AF2-pLDDT performs slightly better than the regular AF2-pLDDT with AUC = 0.737 vs. 0.722, but this improvement is not statistically significant (*p*-value>0.05). The improvement for the AF2-RSA is a little bigger, with AUC = 0.785 for the optimized rank version vs. 0.768 for the regular AF2-RSA, and this difference is statistically significant (*p*-value<0.05). This suggests that AF-RSA based approach to the disorder prediction could be further improved by reranking the predicted models in a way that reflects their capability for the intrinsic disorder prediction.

The overall best disorder predictor is flDPnn, which agrees with the results in CAID [30], [78]. We find that flDPnn’s predictions are statistically better than the AF2-RSA approach, with AUC = 0.814 vs. 0.768 (*p*-value<0.05) and F1 = 0.46 vs. 0.34 (*p*-value<0.05). Overall, four disorder predictors perform better than AF2-RSA, including flDPnn, flDPlr (a version of flDPnn that uses a logistic regression model instead of the deep neural network), rawMSA, and Espritz-D. The results in ref. [47] are similar and show that AF2-RSA is ranked sixth after flDPnn, flDPlr, rawMSA, ESpritz-D and DisoMine. The slight difference stems from the fact that we use the entire CAID dataset while that study uses about 76% of the CAID proteins. The four methods that were published after CAID experiment was completed, IDPseq2seq [71], Metapredict [72], ODiNPred [70], and RFPR [73] perform modestly well with AUCs of 0.754, 0.746, 0.734, and 0.721, respectively. Using the AUC values, we find that AF2-RSA is statistically better than Metapredict, ODiNPred and RFPR (*p*-value<0.05) while the overall best flDPnn outperforms the four tools (*p*-value<0.05).

The top five predictors of disorder (flDPnn, flDPlr, rawMSA, ESpritz-D and AF2-RSA) are characterized by a wide spectrum of runtime values. CAID evaluated the runtime and the corresponding median per-protein values range between 8 s for ESpritz-D, 20 s for flDPnn and flDPlr, and about 300 s for rawMSA [47]. We measured the per-protein runtime for AF2-RSA, which has the median value of 1270 s, with 5th and 95th percentile runtimes of 980 and 3850 s, respectively. This includes the median time of about 980 s to produce multiple alignment (5th percentile of 810 s and 95th percentile of 1870 s) with the remaining runtime spent on encoding the network inputs from the alignment and processing these inputs through the deep network. While we use a different hardware architecture than CAID, which means that our estimate should not be directly compared to the CAID’s results, the magnitudes of the differences are so substantial that we argue that AF2-RSA is at least 50 times slower than the better performing flDPnn and ESpritz-D methods. However, we note that pre-computed AF2 predictions are available for millions of proteins [36], which effectively nullifies the runtime constrains as long as the protein of interest is included in the corresponding database.

Moreover, Table 3 provides coverage values that quantify how many proteins from the CAID datasets were successfully predicted by a given tool, rounded to a nearest percentage point. AF2-RSA failed to produce predictions for about 2% of the test proteins, compared to flDPnn, flDPlr, rawMSA and ESpritz-D that secure the 100% coverage. The lowest coverage of about 94% is for SPOT-Disorder2, which is limited to predicting proteins with sequences shorter than 750 amino acids. This aspect again gives a slight advantage to the modern disorder predictors that provide higher levels of coverage.

Altogether, our analysis suggests that the best disorder predictors outperform the AF2-based disorder predictions by a substantial margin, are substantially faster, and provide a slightly higher coverage.

### 3.2 Comparative evaluation of the residue-level disorder predictions for different types of disordered proteins

We investigate whether predictive quality varies across different types of disordered proteins including those that have only short IDRs (shortIDR dataset), that have at least one long IDR (longIDR dataset); that do not have IDRs at the sequence termini (non-terminusIDR dataset), and disordered proteins with binding IDRs (bindingIDR dataset). Table 4 summarizes these results while using AUC values to quantify the predictive performance.

**Table 4:** Comparative assessment of the AUC values for the residue-level disorder predictions for datasets that consider short IDRs (shortIDR), long IDRs (longIDR), binding IDRs (bindingIDR), and proteins with no IDRs at the sequence termini (non-terminusIDR). We sort predictors by their area under receiver operating characteristic curve (AUC) values on the CAID dataset (Table 3). We assess statistical significance of the differences when compared with the top-ranked flDPnn, AF2-RSA, and AF2-pLDDT, which we highlight using bold font; we show results next to the measured metric using the x|y|z format where x denotes that flDPnn is significantly better (+), worse (-), and not different (=) than the results from a given predictor at p-value = 0.05; y compares against AF2-RSA; and z compares against AF2-pLDDT.

**Predictor**	**longIDR**	**shortIDR**	**bindingIDR**	**non-terminusIDR**
**flDPnn**	**0.824 /-/-**	**0.755 /-/-**	**0.795 /-/-**	**0.744 /-/-**
flDPlr	0.805 + /-/-	0.728 + /-/-	0.767 + /- /-	0.724 + /= /-
AF2-RSAoptimized-rank	0.807 + /-/-	0.669 + /-/-	0.739 + /-/-	0.710 + /= /-
RawMSA	0.805 + /-/-	0.660 + /= /-	0.731 + /-/-	0.687 + /= /=
Espritz-D	0.795 + /= /-	0.661 + /-/-	0.739 + /-/-	0.655 + /= /=
**AF2-RSA**	**0.793 + / /-**	**0.653 + / /-**	**0.721 + / /-**	**0.690 + / /-**
DisoMine	0.784 + /+ /-	0.676 + /-/-	0.721 + /= /-	0.652 + /= /=
SPOT-Disorder2	0.782 + /+ /-	0.658 + /= /-	0.707 + /+ /-	0.660 + /= /=
AUCpred	0.768 + /+ /-	0.681 + /-/-	0.688 + /+ /-	0.684 + /= /=
SPOT-Disorder-Single	0.774 + /+ /-	0.641 + /+ /-	0.689 + /+ /-	0.677 + /= /=
IDPseq2seq	0.773 + /+ /-	0.644 + /+ /-	0.676 + /+ /-	0.669 + /= /=
AUCpred-np	0.766 + /+ /-	0.668 + /-/-	0.683 + /+ /-	0.673 + /= /=
Metapredict	0.761 + /+ /-	0.664 + /-/-	0.690 + /+ /-	0.675 + /= /=
SPOT-Disorder1	0.765 + /+ /-	0.628 + /+ /=	0.662 + /+ /-	0.663 + /= /=
IUPred-short	0.755 + /+ /-	0.666 + /-/-	0.702 + /+ /-	0.679 + /= /=
AF2-pLDDToptimized-rank	0.753 + /+ /-	0.655 + /= /-	0.663 + /+ /-	0.689 + /= /-
IUPred-long	0.759 + /+ /-	0.603 + /+ /+	0.701 + /+ /-	0.673 + /= /=
ODiNPred	0.758 + /+ /-	0.622 + /+ /+	0.690 + /+ /-	0.669 + /= /=
VSL2B	0.750 + /+ /-	0.619 + /+ /+	0.673 + /+ /-	0.676 + /= /=
**AF2-pLDDT**	**0.739 + /+ /**	**0.638 + /+ /**	**0.642 + /+ /**	**0.664 + /= /=**
RFPR	0.750 + /+ /-	0.558 + /+ /+	0.632 + /+ /+	0.645 + /= /+
DISOPRED3	0.722 + /+ /+	0.580 + /+ /+	0.620 + /+ /+	0.634 + /+ /+
DisEMBL-465	0.698 + /+ /+	0.643 + /+ /-	0.637 + /+ /=	0.632 + /+ /+
DisEMBL-HL	0.659 + /+ /+	0.676 + /-/-	0.608 + /+ /+	0.592 + /+ /+

The predictive quality varies rather considerably between these different types of IDPs. Proteins with long IDRs are the easiest to predicts, where an average AUC of the top four disorder predictors is 0.807, AF2-RSA’s AUC is 0.793 and the average AUC across all methods that exclude the optimized rank versions of AF2 is 0.763. We speculate that a potential explanation for that is that AF2-RSA and disorder predictors, which utilize a sliding window approach (i.e., disordered status of the residue in the middle of a sequence segment/window is predicted using information about all residues in that window), benefit from a strong signal that long IDRs provide. In other words, for long IDRs many/majority of residues in a window are disordered, allowing predictive models to more easily differentiate such window from a window that covers structured residues. The IDPs with binding IDRs are the second easiest to predict, with the corresponding AUCs of 0.758 (the top 4 average), 0.721 (AF2-RSA), and 0.690 (all average), respectively. The binding IDRs typically fold upon binding, and some can fold into multiple different conformation depending on the particular ligand that they interact with [79], [80]. This arguably makes them more similar to structured regions when compared to IDRs that do not fold, which in turn should make binding IDRs harder to predict. One plausible explanation why proteins with binding IDRs are predicted with relatively high accuracy is that these IDRs are also rather long (Table 2). This is in contrast with the other two classes of IDPs, which include much shorter IDRs (Table 2) and which are substantially more difficult to predict accurately, with AUCs mostly below 0.7 (Table 4). More specifically, AUC for the IDPs that lack IDRs at the termini are 0.703 (top 4 average), 0.690 (AF2-RSA), and 0.669 (all average); and for IDPs with short IDRs they are 0.701, 0.653, and 0.652, respectively. We note that these trends are consistent across the AF2-based predictions and the results generated by the disorder predictors. The most accurate predictions across all four types of IDPs are secured by flDPnn, which is consistently statistically better than all other tools (*p*-value<0.05). The flDPnn’s AUCs range between 0.824 for IDPs with the long IDRs and 0.744 for the IDP with the non-terminus IDRs.

### 3.3 Comparative evaluation of the protein-level disorder content predictions

We study accuracy of the disorder predictors and AF2 in the context of estimating the per-protein disorder content (Table 5). We find that the mean absolute errors (MAEs) and Spearmen correlation coefficients (SCCs) vary considerably between the predictors. The best results are produced by flDPnn, with MAE = 0.152 and relatively high correlation of 0.59. These predictions are statistically better than the result of all other methods (*p*-value<0.05), except for DisEMBL that produces only slightly higher MAE of 0.161. Interestingly, both versions of DisEMBL and IUPred-short obtain low values of MAE and relatively high values of SCC. This is consistent with observations in ref. [49] where authors applied a different dataset. Moreover, we observe that both AF2-RSA and AF2-pLDDT underperform relative to their residue-level predictions. In particular, AF2-RSA obtains near zero correlation while AF2-pLDDT has a low negative correlation. These low correlations mean that the putative disorder is distributed across proteins in a way that does not correlate with the amount of native disorder. This is in line with the observations from ref. [47], which observed that AF2-pLDDT under-predicts disorder while AF2-RSA over-predicts disorder. Overall, correlations between the quality of the binary residue-level predictions (F1 and MCC in Table 3) and the protein-level content predictions that are derived from these binary predictions (MAE and SCC in Table 5) over the considered predictors are modest, at around − 0.65 for MAE (i.e., negative since lower errors are better) and 0.55 for SCC. This means that the best residue-level predictions not necessarily convert into the best content predictions. Examples include IUPred-short and DisEMBL that perform relatively poorly at the residue level while generating rather accurate content prediction vs. SPOT-Disorder2 and AF2-RSA that produce accurate residue-level predictions while securing relatively high MAE> 0.2 and low SCC< 0.25 for the protein-level content predictions.

**Table 5:** Comparative assessment of the protein-level disorder content predictions on the CAID dataset. We calculate the putative content values as the fraction of the binary predictions of disorder that are established using the threshold that calibrates predictions across methods, and which results in a correct number of predicted disordered residues over the entire CAID dataset. We sort predictors by their area under receiver operating characteristic curve (AUC) values on the CAID dataset (Table 3). We assess statistical significance of the differences when compared with the top-ranked flDPnn, AF2-RSA, and AF2-pLDDT, which we highlight using bold font; we show results next to the measured metric using the x|y|z format where x denotes that flDPnn is significantly better (+), worse (-), and not different (=) than the results from a given predictor at *p*-value = 0.05; y compares against AF2-RSA; and z compares against AF2-pLDDT. Acronyms: Mean Squared Error (MAE) and the Spearman Correlation Coefficients (SCC).

**Predictor**	**MAE**	**SCC**
**flDPnn**	**0.152 /-/-**	**0.589 /-/-**
flDPlr	0.180 + /-/-	0.521 + /-/-
AF2-RSAoptimized-rank	0.211 + /= /-	0.092 + /= /-
RawMSA	0.186 + /-/-	0.230 + /-/-
Espritz-D	0.212 + /= /-	0.477 + /-/-
**AF2-RSA**	**0.213 + / /-**	**0.084 + / /-**
DisoMine	0.195 + /= /-	0.478 + /-/-
SPOT-Disorder2	0.206 + /= /-	0.242 + /-/-
AUCpred	0.195 + /= /-	0.242 + /-/-
SPOT-Disorder-Single	0.197 + /= /-	0.237 + /-/-
IDPseq2seq	0.211 + /= /-	0.173 + /= /-
AUCpred-np	0.188 + /-/-	0.234 + /-/-
Metapredict	0.181 + /-/-	0.300 + /-/-
SPOT-Disorder1	0.232 + /= /-	0.129 + /= /-
IUPred-short	0.172 + /-/-	0.350 + /-/-
AF2-pLDDToptimized-rank	0.252 + /+ /+	-0.339 + /+ /=
IUPred-long	0.197 + /= /-	0.205 + /-/-
ODiNPred	0.199 + /= /-	0.167 + /-/-
VSL2B	0.201 + /= /-	0.175 + /-/-
**AF2-pLDDT**	**0.246 + /+ /**	**-0.341 + /+ /**
RFPR	0.244 + /= /=	0.100 + /-/-
DISOPRED3	0.240 + /= /=	-0.088 + /+ /-
DisEMBL-465	0.161 = /-/-	0.414 + /+ /-
DisEMBL-HL	0.163 = /-/-	0.384 + /+ /-

### 3.4 Comparative evaluation of the protein-level predictions of fully disordered proteins

Following the assessment in CAID [30], we consider protein-level prediction of the fully disordered proteins which are defined as IDPs with a very high disorder content. Since there are no well-defined cut-offs, we consider three scenarios where fully disordered proteins are defined based on the disorder content> 0.99,> 0.90 and> 0.80, similar to what was done in CAID. We summarize these results in Table 6. The best predictions are secured by flDPnn, which obtains F1 of about 0.90 and sensitivity of 0.83 across the three scenarios. Its predictions are also statistically better than the results of all other tools (*p*-value<0.05). Similar to the residue-level predictions of disorder (Table 3), four disorder predictors (flDPnn, flDPlr, rawMSA and ESpritz-D) are significantly better than AF-RSA across the three definitions of the fully disordered proteins and both metrics (*p*-value<0.05). Moreover, AF2-RSA is statistically better than AF2-pLDDT (*p*-value<0.05), where the latter predictor generates results at a near random levels. Overall, the residue-level F1 values (Table 3) are highly correlated with the F1 values for the prediction of fully disorder proteins (Table 6), with correlations at about 0.76 across the three scenarios. Moreover, results across the three fully disordered protein definitions are highly correlated when considering both F1 and sensitivity (correlations of 0.99). Altogether, we find that AF2-RSA provides modestly accurate predictions of fully disordered proteins, AF2-pLDDT should not be used to identify fully disordered proteins, and several disorder predictors outperform AF2-RSA.

**Table 6:** Comparative assessment of the protein-level predictions of fully disordered proteins (FDPs) on the FDP99, FDP90, and FDP80 datasets. We sort predictors by their area under receiver operating characteristic curve (AUC) values on the CAID dataset (Table 3). We assess statistical significance of the differences when compared with the top-ranked flDPnn, AF2-RSA, and AF2-pLDDT, which we highlight using bold font; we show results next to the measured metric using the x|y|z format where x denotes that flDPnn is significantly better (+), worse (-), and not different (=) than the results from a given predictor at *p*-value = 0.05; y compares against AF2-RSA; and z compares against AF2-pLDDT.

**Predictor**	**FDP99 dataset**		**FDP90 dataset**		**FDP80 dataset**	
	**F1**	**sensitivity**	**F1**	**sensitivity**	**F1**	**sensitivity**
**flDPnn**	**0.906 /-/-**	**0.829 /-/-**	**0.911 /-/-**	**0.839 /-/-**	**0.898 /-/-**	**0.827 /-/-**
flDPlr	0.872 + /-/-	0.772 + /-/-	0.878 + /-/-	0.783 + /-/-	0.862 + /-/-	0.767 + /-/-
AF2-RSAoptimized-rank	0.518 + /+ /-	0.350 + /+ /-	0.555 + /+ /-	0.384 + /+ /-	0.534 + /+ /-	0.366 + /+ /-
RawMSA	0.799 + /-/-	0.666 + /-/-	0.804 + /-/-	0.672 + /-/-	0.762 + /-/-	0.619 + /-/-
Espritz-D	0.864 + /-/-	0.760 + /-/-	0.866 + /-/-	0.764 + /-/-	0.843 + /-/-	0.731 + /-/-
**AF2_RSA**	**0.557 + / /-**	**0.386 + / /-**	**0.594 + / /-**	**0.423 + / /-**	**0.567 + / /-**	**0.398 + / /-**
DisoMine	0.817 + /-/-	0.690 + /-/-	0.826 + /-/-	0.707 + /-/-	0.823 + /-/-	0.707 + /-/-
SPOT-Disorder2	0.665 + /-/-	0.498 + /-/-	0.681 + /-/-	0.517 + /-/-	0.662 + /-/-	0.498 + /-/-
AUCpred	0.706 + /-/-	0.546 + /-/-	0.711 + /-/-	0.555 + /-/-	0.692 + /-/-	0.537 + /-/-
SPOT-Disorder-Single	0.635 + /-/-	0.465 + /-/-	0.650 + /-/-	0.482 + /-/-	0.619 + /-/-	0.451 + /-/-
IDPseq2seq	0.627 + /-/-	0.457 + /-/-	0.640 + /-/-	0.470 + /-/-	0.616 + /-/-	0.445 + /-/-
AUCpred-np	0.579 + /= /-	0.408 + /= /-	0.585 + /= /-	0.415 + /= /-	0.567 + /= /-	0.399 + /= /-
Metapredict	0.657 + /-/-	0.489 + /-/-	0.672 + /-/-	0.506 + /-/-	0.645 + /-/-	0.479 + /-/-
SPOT-Disorder1	0.523 + /= /-	0.354 + /= /-	0.549 + /+ /-	0.378 + /+ /-	0.529 + /+ /-	0.362 + /+ /-
IUPred-short	0.605 + /-/-	0.434 + /-/-	0.624 + /-/-	0.454 + /-/-	0.609 + /-/-	0.441 + /+ /-
AF2-pLDDToptimized-rank	0.023 + /+ /-	0.012 + /+ /+	0.028 + /+ /+	0.014 + /+ /+	0.027 + /+ /+	0.014 + /+ /+
IUPred-long	0.645 + /-/-	0.477 + /-/-	0.662 + /-/-	0.495 + /-/-	0.641 + /-/-	0.474 + /+ /-
ODiNPred	0.524 + /= /-	0.354 + /= /-	0.518 + /+ /-	0.349 + /+ /-	0.509 + /+ /-	0.341 + /+ /-
VSL2B	0.607 + /-/-	0.436 + /-/-	0.619 + /-/-	0.448 + /-/-	0.590 + /= /-	0.419 + /= /-
**AF2_pLDDT**	**0.069 + /+ /**	**0.036 + /+ /**	**0.069 + /+ /**	**0.036 + /+ /**	**0.063 + /+ /**	**0.033 + /+ /**
RFPR	0.479 + /+ /-	0.315 + /+ /+	0.505 + /+ /=	0.338 + /+ /-	0.467 + /+ /-	0.305 + /+ /-
DISOPRED3	0.347 + /+ /-	0.210 + /+ /+	0.361 + /+ /-	0.220 + /+ /-	0.331 + /+ /-	0.198 + /+ /-
DisEMBL-465	0.614 + /+ /-	0.443 + /-/-	0.613 + /= /-	0.443 + /= /-	0.589 + /= /-	0.421 + /= /-
DisEMBL-HL	0.572 + /= /-	0.400 + /= /-	0.557 + /= /-	0.387 + /= /-	0.533 + /= /-	0.366 + /= /-

### 3.5 Sequence-derived markers identify proteins for which AF2-RSA outperforms disorder predictors

Our results suggest that several disorder predictors are more accurate and faster than AF2-RSA when tested on large datasets of proteins. However, quality of disorder predictions varies widely across individual proteins [50], which motivated us to investigate whether AF2-RSA-based predictions could be competitive for certain types of disordered proteins. In other words, we attempt to identify sequence-derived markers that can be used to identify proteins for which AF2 predicts disorder as accurately or better than the best disorder predictors. We measure predictive performance with AUC and use the CAID dataset. We have to exclude 45 fully disordered proteins for which we cannot compute AUC since they do not include native structured residues. We use the sequence, disorder predicted from sequence with the most accurate flDPnn [60], protein-binding IDRs and coiled-coil regions predicted from sequence with popular ANCHOR [81] and DeepCoil [82] methods, respectively, to derive several diverse markers. The inclusion of the coiled-coils is motivated by an observation that they are often disordered and may transition into the structured state via intramolecular interactions [83]. We consider eight markers: 1) sequence length; 2) putative disorder content; 3) putative content of protein-binding IDRs; 4) number of putative IDRs; 5) maximal length of putative IDRs; 6) putative content of coiled-coil regions; 7) distance of putative IDRs to a closest terminus (proxy for presence of putative IDRs at the terminus); and 8) a composite score that considers distance to terminus and content of the putative disorder. We calculate the composite score as sum of the distances of putative disordered residues to the nearest terminus divided by the sequence length. Low values of this score indicate that the disorder content is low and/or disorder is located at the termini. We consider IDRs that are defined as sequence segments of at least 4 consecutive putative disordered residues. We divide the CAID dataset into two subsets: proteins for which AF2-RSA is competitive (i.e., it generates highly accurate predictions that are statistically as accurate as the results of the best disorder predictors vs. proteins for which AF2-RSA is statistically outperformed by disorder predictions or generates lower accuracy predictions. The first group includes 195 proteins for which AF2-RSA’s AUC>0.814 (i.e., AUC is greater than an expected value of a highly accurate AUC that equals to the overall/dataset-level AUC of the best performing flDPnn from Table 3) and for which protein-level AUC of AF2-RSA is within the 5% confidence interval of the AUCs of the considered 19 disorder predictors. This means that AF2-RSA generates competitive disorder predictions for about one-third of the IDPs from the CAID dataset. The remaining proteins constitute the second subset.

**Fig. 2:**
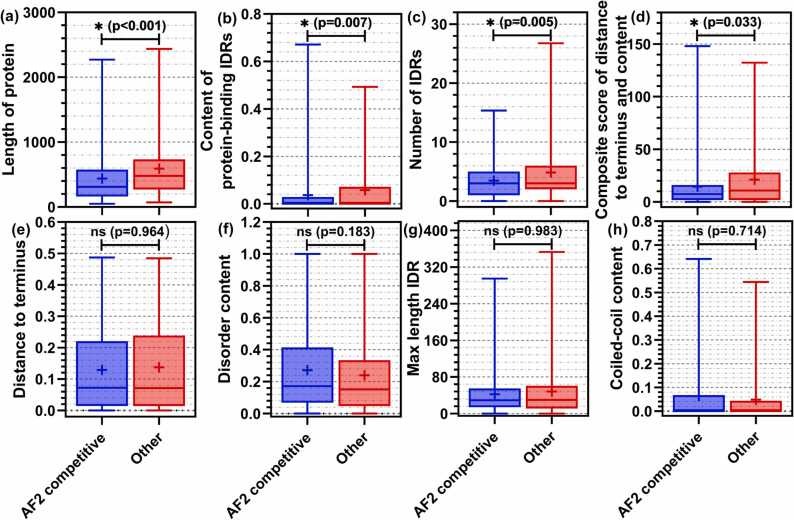
Comparison of the sequence-derived markers between proteins for which AF2-RSA generates competitive predictions (blue box plots) vs. proteins for which AF2-RSA is statistically outperformed by disorder predictions or generates lower accuracy predictions (red box plots). The markers include: (panel a) sequence length; (panel b) putative content of binding IDRs; (panel c) number of putative IDRs; (panel d) composite score of distance to terminus and content of the putative disorder; (panel e) distance of putative IDRs to a closest terminus; (panel f) putative disorder content; (panel g) maximal length of putative IDRs; and (panel h) putative content of coiled-coils. Box plots represent distributions of the marker values in a given protein set where we show the 5th, 25th, 50th (median), 75th and 95th percentiles and where cross represents the average. Statistical significance of differences is annotated above the box plots: ns means difference is not significant; * means significant.

We compare values of the eight markers between the two protein sets in Fig. 2. We find that the putative disorder and coiled-coils content values, inclusion of long IDRs, and distance of IDRs to the termini (Fig. 2e, f, g and h) cannot be used to reliably identify IDPs for which AF2-RSA produces accurate results (*p*-values>0.18). Interestingly, the composite score that combines putative disorder content and distance of disorder from termini (Fig. 2d) is statistically significant (*p*-value = 0.03). This marker reveals that AF2-RSA produces accurate predictions of disorder for IDPs that have relatively low disorder content and where this disorder is located at or close to the sequence termini. This can be explained by the fact that AF2 was trained using structures from PDB that have disorder which is often located at the termini and that have relatively low disorder content [84]. Three other markers are also statistically significant. AF-RSA is biased to generate accurate disorder predictions for IDPs that have short sequences (*p*-value<0.001; Fig. 2a), that have relatively few putative IDRs (*p*-value = 0.005; Fig. 2c) and low (near-zero) content of protein-binding IDRs (*p*-value = 0.007; Fig. 2b). We think that the first two markers can be explained by the fact that PDB structures typically cover short protein chains and that these sequences typically have relatively few IDRs [66], [85]. Moreover, the third marker is reinforced by a recent analysis that suggests that AF2′s predictions suffer substantially lower quality for multimers [86]. We use these four statistically significant markers to identify proteins for which AF2-RSA generates very accurate disorder predictions. We select proteins for which sequence length (Fig. 2a), content of protein binding IDRs (Fig. 2b), number of IDRs (Fig. 2c), and the composite score (Fig. 2d) are below the median values from the blue box plots. This dataset has 106 proteins, which corresponds to approximately 20% of the IDPs in the benchmark dataset. We find that AF2-RSA secures AUC = 0.81 for these proteins, outperforming the best disorder predictors that are overall shown to be more accurate than AF2-RSA (Table 3), including flDPnn (AUC = 0.79), flDPlr (AUC = 0.78), rawMSA (AUC = 0.78), and Espritz-D (AUC = 0.77). The differences in the AUC values between the AF2-RSA predictions and the results of these best disorder predictors for this protein set are statistically significant (*p*-value<0.01). Altogether, we conclude that AF2-RSA should be used to identify disorder for smaller proteins that lack protein-binding IDRs, and which have relatively few short IDRs that are located at the sequence termini. The other IDPs should be predicted using modern disorder predictors.

## 4 Summary and conclusions

Similar to the previous studies [46], [47], [87], we find that AF2-RSA outperforms AF2-pLDDT and that several modern disorder predictors outperform AF2-RSA by a statistically significant margin. Moreover, we empirically demonstrate that some of these accurate disorder predictors are also substantially faster and provide a slightly higher coverage. We also provide several new insights. Using the AF2-RSAoptimized-rank approach, we empirically observe that AF-RSA could be modestly improved by reranking the AF2 predicted models in a way that better reflects their potential for the disorder prediction. We find that several disorder predictors outperform AF2-RSA’s ability to predict fully disordered proteins and that AF2-pLDDT should not be used to predict these proteins. Both AF2-RSA and AF2-pLDDT provide poor predictions of disorder content while several disorder predictors, such as flDPnn, IUPred-short and DisEMBL accurately predict the content values. Our empirical analysis that relies on several sequence-derived markers suggests that AF2-RSA outperforms disorder predictors for about 20% of IDPs that have short sequences absent of disordered protein-binding regions and which have relatively few IDRs that are preferably located at the sequence termini. We suggest that AF2 should be used to predict disorder for these proteins since these results can be produced as a byproduct of the structure predictions, incurring only a small additional computational cost. However, disorder predictors, such as flDPnn, flDPlr, rawMSA and Espritz-D, should be used to make disorder predictions for the other disordered proteins.

We also identify a substantial variability in the predictive quality across different types of disordered proteins. In particular, we observe that some IDPs are substantially harder to predict accurately for AF2-RSA, AF2-pLDDT and disorder predictors, including IDPs that lack IDRs at the sequence termini and those that have only short IDRs. Recent literature offers additional insights concerning limitations of AF2. The use of AF2 could lead to misinterpretations of predicted “structures” of IDRs in the context of the sequence-ensemble-function relationships that are characteristic for the interactomes of disordered proteins [88]. Moreover, AF2′s predictions were also shown to have lower quality for the proteins with dynamic structures [89], [90] and multimers [86].

In recent years, the disorder prediction field has moved towards prediction of functional types of IDRs [91], [92], [93], [94]. The main focus is on the regions that interact with ligands that include peptides, proteins, DNA, RNA and lipids [91], [92], [95], however, some tools also predict disordered linkers [96], [97]. There are over three dozen of predictors of binding IDRs, with majority of them targeting protein and peptide binding IDRs [91]. Example popular methods include ANCHOR [81], [98], [99], MoRFpred [100**]**, MoRF_chibi_ [101], [102], and DISOPRED3 [67] that predict protein and peptide binding IDRs; DisoRDPbind that predicts DNA, RNA and protein-binding IDRs; and SLiMFinder [103**]** and SLiMSearch [104], [105], [106] that predict short linear sequence motifs (SLiMs) that are often involved in the protein-protein and protein-nucleic acids interactions. We also note the two recently released tools that predict lipid binding IDRs: MemDis [107**]** and DisoLipPred [108**]**, and the DEPICTER webserver that predicts multiple types of functional IDRs [109], [110]. Interestingly, AF2, the most accurate disorder predictors [31], and some of the predictors of binding IDRs rely on the deep neural network models [108], [111], [112], [113], [114], [115]. This suggests that deep networks are useful for both protein structure and intrinsic disorder predictions.

## CRediT authorship contribution statement

**Bi Zhao**: Data curation, Formal analysis, Investigation, Visualization, Writing − original draft. **Lukasz Kurgan**: Conceptualization, Formal analysis, Funding acquisition, Investigation, Project administration, Resources, Supervision, Visualization, Writing − original draft, Writing − revised draft. **Sina Ghadermarzi**: Data curation, Formal analysis.

## Conflicts of interests

The authors declare no conflicts of interest.
